# Cataract surgery and the risk of developing recurrence or progression of diabetic macular edema

**DOI:** 10.1371/journal.pone.0328874

**Published:** 2025-08-18

**Authors:** Mirinae Kim, Young-Gun Park, Young-Hoon Park

**Affiliations:** Department of Ophthalmology and Visual Science, College of Medicine, The Catholic University of Korea, Seoul, Republic of Korea; University of Colorado Denver School of Medicine, UNITED STATES OF AMERICA

## Abstract

**Purpose:**

To determine whether uneventful cataract surgery in patients with diabetic macular edema (DME) affects the course of the disease, and its relationship with postoperative clinical outcomes in real-world settings.

**Materials and Methods:**

This retrospective cohort study included patients of center-involving DME and a prior history of periocular injection in the operative eye before cataract surgery with a follow-up of at least 6 months. Patients were assigned to active and inactive DME group according to preoperative status. Patients’ clinical outcome measurements before and after cataract surgery were compared between the groups. Cox-proportional hazards model was performed to identify risk factors of DME recurrence or progression after cataract surgery.

**Results:**

The study included 153 eyes in 153 patients. No significant differences were observed in the trend of the groups’ clinical outcomes, including best-corrected visual acuity, central macular thickness and central choroidal thickness (P = .763,.872, and.127, respectively). Patients with higher HbA1c were more likely to develop recurrence or progression of DME after cataract surgery (hazard ratio = 1.407, *P* = .039).

**Conclusion:**

The clinical outcomes following cataract surgery in patients with inactive or actively treated DME did not show significant differences at any postoperative period. Recurrence or progression of DME after cataract surgery was found to be associated with high HbA1c. Clinicians do not have to delay the cataract surgery in patients with DME who have good glycemic control and are undergoing treatment as needed.

## Introduction

Diabetes mellitus (DM) is associated with an approximately 5-fold increased prevalence of cataract with cortical and posterior subcapsular opacities, compared with the non-diabetic population [1–5]. In the Wisconsin Epidemiologic Study of Diabetic Retinopathy, cataract was a significant cause of legal blindness, second only to proliferative diabetic retinopathy, in younger-onset patients with diabetes, and the most frequent cause of visual loss in older-onset patients with diabetes [6]. Given the epidemiologic trends of cataracts and DM, ophthalmologists will likely encounter patients with comorbidities requiring cataract surgery. In deciding the appropriate timing of cataract surgery, clinicians have many considerations compared to patients without DM who have cataracts. Major considerations include the presence of macular edema or vitreous/preretinal hemorrhage and glycemic control status.

In our clinical practice, determining the appropriate timing for cataract surgery in patients with a history of diabetic macular edema (DME) poses substantial challenges. Several studies have evaluated the impact of cataract surgery on DME exacerbation in patients with diabetes [7–11]. Uneventful cataract surgery is also associated with an increased risk of developing macular edema or worsening of the preexisting one due to alterations in the aqueous levels of angiogenic and anti-angiogenic factors following the surgical procedure [7–9]. According to DRCR network reports, in eyes with diabetic retinopathy without center-involving DME, any history of DME may increase the risk of developing DME after cataract surgery [10]. However, Starr et al. recently reported significant visual acuity improvements after cataract surgery in patients with DME who were actively managed with perioperative intravitreal anti-VEGF injections [11]. If DME is a risk factor for developing macular edema after cataract surgery, the question arises: should cataract surgery be delayed in patients receiving treatment for DME?

This study aimed to determine whether uneventful cataract surgery in patients with DME affects the course of the disease, and its relationship with postoperative clinical outcomes in real-world settings.

## Materials and methods

### Study population

The study protocol was approved by the Institutional Review Board of the Catholic University of Korea (approval number: KC24RISI0625) and adhered to the tenets of the Declaration of Helsinki. The requirement for written informed consent was waived because of the retrospective nature of this study. Data were accessed for this study from September 20th, 2024 to October 10th, 2024 in accordance with IRB approval.

Our study was a retrospective cohort analysis of consecutive cataract surgeries performed on patients with DME at Seoul St. Mary’s Hospital between January 2013 and December 2021. Inclusion criteria included a diagnosis of center-involving DME and a prior history of periocular injection in the operative eye before cataract surgery with a follow-up of at least 6 months. Patients who had received periocular injection in the operative eye within 6 months before surgery were allocated to the active DME group, while those who had received the same injection in the operative eye more than 6 months before surgery were allocated to the inactive DME group. We also included age- and sex-matched patients with DME who did not undergo cataract surgery as a control group. Exclusion criteria included (1) complicated cataract surgery or postoperative complication including endophthalmitis; (2) history of intravitreal injection treatment other than diabetes (i.e., retinal vein occlusion, exudative age-related macular degeneration); (3) history of uveitis; (4) presence of glaucoma or other concomitant retinal diseases that could affect central macular thickness; and (5) patients who did not complete 6 months of follow-up.

### Patient evaluation and image analysis

Patient characteristics, including age, sex, follow-up duration, medical history, history of panretinal photocoagulation or ocular surgeries, and blood test results, including HbA1c, eGFR, and creatine, were retrieved from medical records. All patients underwent comprehensive baseline and follow-up ocular examinations. Best-corrected visual acuity (BCVA) was measured using a standard Snellen chart and converted to logarithm of the minimal angle of resolution (logMAR) units before analysis. Slit-lamp examination and fundus examination was performed using a Haag-Streit BQ 900 slit lamp (Haag-Streit AG, Köniz, Switzerland). Intraocular pressure (IOP) was measured by pneumatic tonometry (TX-20P, Canon, Japan). Optical coherence tomography (OCT) images were recorded at preoperative, 1-month, 3-month, 6-month, and 12-month intervals postoperatively. Eyes were scanned using either a spectral domain OCT (Spectralis HRA OCT; Heidelberg Engineering, Heidelberg, Germany) or a swept-source OCT (DRI-OCT; Topcon Corp, Tokyo, Japan). The same OCT device was used for each patient throughout the follow-up period. Central macular thickness (CMT) and central choroidal thickness (CCT) were measured using either a circular map analysis provided by the spectral domain OCT software or the macular volume scan of the swept-source OCT device.

We analyzed the recurrence or progression of DME as a composite outcome. Recurrence or progression of DME after cataract surgery was defined according to the categorization outlined in the DRCR network report [10]. The definition of recurrence or progression of DME at follow-up was as follows; study eye meeting any 1 of the following (1) ≥1 inner subfield (ISF) thickness ≥310 μm at 16 week and the corresponding ISF thickness increased ≥1 logOCT unit from baseline to 16 week or ≥1 outer subfield (OSF) thickness ≥290 μm at 16 week and the corresponding OSF thickness increased ≥1 logOCT unit from baseline to 16 week; (2) ≥1 ISF thickness increased ≥2 logOCT units from baseline to 16 week or ≥1 OSF thickness increased ≥2 logOCT units from baseline to 16 week; or (3) any treatment for DME or CME other than topical eyedrops received after surgery and criterion 1 or 2 met prior to starting treatment.

### Statistical analysis

Categorical data were expressed as absolute numbers, and continuous data as mean ± standard deviation (95% confidence interval). Kruskal-Wallis test was used to compare baseline characteristics between the three groups. The repeated measures ANOVA with Bonferroni post-hoc correction was used to compare the patients’ clinical outcome measurements before and after cataract surgery. Cox proportional hazards models were used to identify the factors on DME recurrence or progression after cataract surgery. Hazard ratios (HRs) were estimated using univariable Cox regression analysis to evaluate the association between individual clinical variables and DME recurrence or progression. The cumulative probabilities of DME recurrence or progression after cataract surgery were assessed using Kaplan–Meier analysis. A log-rank test was used to compare the survival rates between the two groups.

All statistical analyses were performed using SPSS for Windows (version 24.0; IBM, Armonk, New York, USA). Kaplan–Meier survival curves were created using GraphPad Prism version 8.4.2 for Windows (San Diego, California, USA). Results with *P*-values less than.05 were considered statistically significant and adjusted *P*-value less than 0.01 was set as the cutoff value in Bonferroni’s post hoc analysis.

## Results

### Demographic and clinical characteristics

This study included 95 eyes of active DME and 58 of inactive DME. The baseline demographics and clinical characteristics of study participants are presented in **Table 1**. The mean age of the patients was 61.06 ± 9.15 years in the active DME group and 61.89 ± 9.40 years in the inactive DME group. The age at the time of cataract surgery and sex distribution were comparable among the groups. No significant differences were observed in HbA1c, serum creatinine level, eGFR, severity of diabetic retinopathy, type of previous DME, and axial length between the two groups (all **P* *> .05). Baseline CMT was 314.09 ± 76.30 µm in active DME group and 278.03 ± 56.07 µm in inactive DME group (*P* = .002). However, the baseline BCVA did not differ significantly between both groups (0.82 ± 0.37 in the active DME group vs 0.74 ± 0.37 in the inactive DME group, *P* = .211). Within 3 months after cataract surgery, recurrence or progression of DME was observed in 50 eyes (52.5%) in the active DME group, and 23 eyes (39.7%) in the inactive DME group, but with no statistical significance observed (*P* = .177).

**Table 1:** Baseline demographics and clinical characteristics of patients with DME.

Variables	Non-cataract surgery	Cataract surgery in active DME	Cataract surgery in inactive DME	*P* value
Patients (n)	64	95	58	
Age at the time of cataract surgery (years)	61.51 ± 9.38	61.06 ± 9.15	61.89 ± 9.40	.589
Male, n (%)	31 (48.4%)	43 (45.3%)	31 (53.4%)	.326
HbA1c	7.22 ± 1.06	7.45 ± 1.30	7.34 ± 0.95	.287
Serum creatinine	1.50 ± 1.42	1.36 ± 1.66	1.58 ± 2.05	.464
eGFR	68.67 ± 25.52	71.60 ± 29.40	65.07 ± 24.97	.545
Severity of diabetic retinopathy				.974
NPDR	38 (59.4%)	62 (65.3%)	38 (65.5%)	
PDR	26 (40.6%)	33 (34.7%)	20 (34.5%)	
Type of previous DME				.522
Cystoid	33 (51.6%)	53 (55.8%)	31 (53.4%)	
DRT	18 (28.1%)	20 (21.1%)	18 (31.0%)	
SSRD	3 (4.7%)	2 (2.1%)	2 (3.4%)	
Combination of above	10 (15.6%)	20 (21.1%)	7 (12.1%)	
History of PRP treatment, n (%)	43 (67.2%)	60 (63.2%)	38 (65.5%)	
SE refraction error (diopters)	−0.22 ± 2.50	−0.57 ± 2.20	−0.90 ± 2.35	.384
Axial length (mm)	–	23.45 ± 0.99	23.62 ± 1.03	.301
Baseline best-corrected visual acuity (logMAR)	0.53 ± 0.22	0.82 ± 0.37	0.74 ± 0.37	.211
Baseline CMT (μm)	299.15 ± 92.59	314.09 ± 76.30	278.03 ± 56.07	.002
Baseline CCT (μm)	277.13 ± 81.25	266.79 ± 63.07	279.29 ± 70.37	.257
Number of injections prior to cataract surgery	–	5.41 ± 3.79	5.21 ± 3.93	.751
Time from last anti-VEGF injection to cataract surgery (mos)	–	2.99 ± 1.67	15.16 ± 3.62	<.001
Recurrence of DME after cataract surgery	–	50 (52.6%)	23 (39.7%)	.177
Mean number of injections in postop 1 year	–	2.43 ± 1.74	1.93 ± 1.84	.093
Follow-up duration (mos)	51.12 ± 25.91	49.51 ± 28.70	48.52 ± 26.81	.833

Data are expressed as mean ± standard deviation (95% confidence interval).

CCT, central choroidal thickness; CMT, central macular thickness; DME, diabetic macular edema; DRT, diffuse retinal thickening; GFR, glomerular filtration rate; NPDR, non-proliferative diabetic retinopathy; PDR, proliferative diabetic retinopathy; PRP, panretinal photocoagulation; SE, spherical equivalent; SSRD, subretinal serous retinal detachment.

### Clinical outcomes after cataract surgery

**Table 2** shows the groups’ clinical outcomes, including BCVA, CMT, and CCT. BCVA showed a significant improvement from 1 month after surgery, with no significant difference in the trend of improvement between the two groups (**P* *= .763). CMT increased at 1 month after cataract surgery, gradually decreased, and tended to return to a level similar to preoperative CMT value at 12 months. No significant difference was observed in the trend between the two groups (**P* *= .872). The mean number of injections in the postoperative period did not differ significantly between the two groups (2.43 ± 1.74 and 1.93 ± 1.84 in the active and inactive DME groups, respectively; *P* = .093). (Table 1) Additionally, no difference was observed in CCT over time and between the two groups (P = .127).

**Table 2:** Outcome measurements in patients with DME after cataract surgery.

	Preop	POD 1M	3M	6M	12M	*P* (time)	*P* (time*group)
BCVA (logMAR)							
Active DME	0.82 ± 0.37	0.48 ± 0.26	0.44 ± 0.26	0.45 ± 0.45	0.44 ± 0.28	*<.001*	
Inactive DME	0.74 ± 0.37	0.43 ± 0.28	0.41 ± 0.25	0.39 ± 0.26	0.39 ± 0.29	*<.001*	
*P* value^a^ (group)	*.211*	*.183*	*.106*	*.176*	*.125*		*.763*
CMT (μm)							
Active DME	314.09 ± 76.30	384.36 ± 143.83	358.55 ± 108.06	330.28 ± 95.44	322.47 ± 99.52	*<.001*	
Inactive DME	278.03 ± 56.07	337.69 ± 101.33	319.41 ± 75.15	298.79 ± 75.83	275.41 ± 61.51	*<.001*	
*P* value^a^ (group)	*.002*	*.020*	*.009*	*.026*	*<.001*		*.872*
CCT (μm)							
Active DME	266.79 ± 63.07	281.11 ± 82.69	282.16 ± 81.56	270.83 ± 78.66	271.38 ± 78.96	*.103*	
Inactive DME	279.29 ± 70.37	273.47 ± 76.99	277.71 ± 76.98	267.07 ± 67.90	275.43 ± 71.08	*.161*	
*P* value^a^ (group)	*.257*	*.562*	*.738*	*.763*	*.750*		*.127*

^a^*P* value by repeated measures ANOVA with Bonferroni post hoc test

BCVA, best-corrected visual acuity; CCT, central choroidal thickness; CMT, central macular thickness; DME, diabetic macular edema

**Fig 1:**
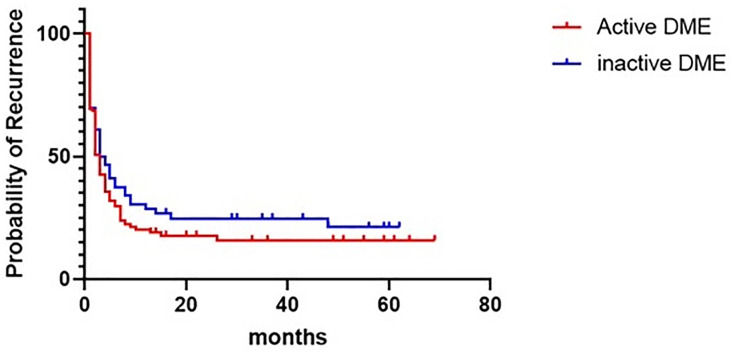
Kaplan–Meier curve of time to recurrence or progression of diabetic macular edema (DME) after cataract surgery (log-rank test, *P* = .228).

**Fig 1** shows the Kaplan–Meier curve of time to recurrence or progression of DME after cataract surgery. No statistical difference was observed between the two groups

(*P* = .228, log-rank test), and the median survival time to recurrence was 6 months in the active DME group and 6.5 months in the inactive DME group.

### Cox proportional hazards model

We subsequently evaluated the risk factors of recurrence or progression of DME after cataract surgery (**Table 3**). The cox proportional hazards model revealed that patients with higher HbA1c were more likely to develop recurrence or progression of DME after cataract surgery (hazard ratio = 1.407, *P* = .039). Age, sex, DM duration, serum creatinine, axial length, severity of DR, previous number of injections, preoperative BCVA, preoperative CMT, or preoperative CCT were not associated with an increased risk of recurrence or progression of DME after cataract surgery.

**TABLE 3:** Cox proportional hazard model for prediction of recurrence or progression of DME after cataract surgery.

	Hazard Ratio	95% CI	*P*
Age	1.000	0.983–1.018	.960
Female sex	1.274	0.880–1.843	.199
DM duration	1.006	0.983–1.030	.611
HbA1c	1.407	0.858–2.482	.039
Creatinine	1.042	0.935–1.160	.459
Axial length	0.950	0.784–1.150	.599
DR grade	1.056	0.727–1.534	.773
Previous number of injections	1.018	0.974–1.065	.425
Group (Active or Inactive)	1.161	0.800–1.685	.433
Preoperative BCVA	1.790	1.067–3.004	.127
Preoperative CMT	0.999	0.996–1.001	.266
Preoperative CCT	1.001	0.999–1.004	.276

BCVA, best-corrected visual acuity; CCT, central choroidal thickness; CMT, central macular thickness; DM, diabetes mellitus; DR, diabetic retinopathy

## Discussion

In this study, we assessed the clinical outcomes in patients with DME undergoing cataract surgery by categorizing DME eyes into active and inactive DME groups based on previous treatment. The clinical outcomes, including BCVA and CMT, demonstrated no inferiority in actively treated DME eyes. Notably, preoperative HbA1c emerged as the single risk factor for the recurrence or progression of DME after uncomplicated cataract surgery.

The relationship between uneventful cataract surgery and DME progression or aggravation has been a subject of debate with conflicting evidence. In a large database study of 81,984 eyes, the risk of developing macular edema after cataract surgery was higher in the presence of any DR, even in eyes without DME [12]. In the DRCR network reports, clinical outcomes of cataract surgery in patients without center-involving DME at the time of cataract surgery [10] and with center-involving DME [13] were reported. In the first report, including those eyes without center-involving DME, any history of DME before cataract surgery increased the risk of developing DME afterward. In the second report, including those eyes with center-involving DME, a small percentage of eyes had substantial VA loss or definitive worsening in central retinal thickening. A recent real-world data by Starr et al. [11] examined the clinical outcomes of cataract surgery in active DME managed with anti-VEGF. The study reported that 46% of patients with DME, actively managed with perioperative intravitreal anti-VEGF injections, developed new or worsening DME but still experienced a significant improvement in VA. In our study population, we found that clinical outcomes of cataract surgery in patients in the inactive and active DME groups were comparable and showed significant visual improvements postoperatively. Our findings suggest that clinicians may not have to delay cataract surgery in patients with DME if actively managed with periocular injection.

In patients with diabetes, cataract surgery may induce macular edema due to increased inflammatory mediators in the aqueous and vitreous humor after surgical manipulation. Shimura et al. [14] and Ching et al. [15] reported that macular thickening after cataract surgery peaks at 4–8 weeks after surgery in patients with diabetes. However, in our study population, CMT peaked at 1 month after cataract surgery. Critical clinical evaluations, including OCT scans at 1 month after surgery, play a vital role in determining the necessity of intravitreal injection treatment. Many studies focused on the efficacy of preoperative or perioperative treatment for DME using intravitreal anti-VEGF or periocular steroid injections [16–22]. Intravitreal injections administered preoperatively, postoperatively, and concomitantly with cataract surgery showed comparable efficacy in preventing the worsening of macular edema. Preoperative intravitreal injections are often initiated 1–2 weeks prior to cataract surgery, and adjunctive use of topical nonsteroidal anti-inflammatory drugs (NSAIDs) is common in patients with diabetes [23–27].

This study has a few limitations. First, its retrospective nature resulted in variable use of perioperative topical eyedrops, including NSAIDs, within the study populations. In patients with diabetes, prophylactic preoperative or postoperative NSAIDs may have a role in preventing macular edema following cataract surgery [24,27]. Second, we did not include patients with diabetes without a history of DME as an additional control group. However, the diverse spectrum of patients with diabetes without a history of DME could introduce additional confounding factors. Third, the intravitreal injections were administered according to the PRN protocol, with treatment decisions and follow-up intervals left to the physician’s judgment. Differentiating new-onset pseudophakic cystoid macular edema and aggravation of DME may also pose challenges. Fourth, there may be selection bias as the study only targeted patients who visited a tertiary care center. Lastly, although the recurrence and progression of DME may represent distinct clinical courses with potentially different risk profiles, we reported them as a combined outcome in our analysis. This decision was made because the number of events in each category was too small to allow for meaningful statistical comparison. Although the differences in recurrence rates and CMT between the two groups did not reach statistical significance, the magnitude of these differences may still carry clinical relevance. Specifically, the recurrence rate was higher in the active group (52%) compared to the non-active group (39%), and the mean difference in CMT between the groups was approximately 50 µm. While these findings were not statistically significant, they suggest a potential trend toward worse anatomical outcomes in eyes with active DME. Further studies with larger sample sizes are needed to confirm the clinical significance of these observations. Despite these limitations, the main strengths of our study lie in the extended postoperative evaluation periods for BCVA and CMT, as well as the analysis of predictive factors for the recurrence or progression of DME after cataract surgery in a real-world setting.

In summary, the clinical outcomes following uneventful cataract surgery in patients with inactive or actively treated DME did not show significant differences at any postoperative period. Recurrence or progression of DME after cataract surgery was found to be associated with high HbA1c. Based on these findings, we concluded that clinicians do not have to delay the cataract surgery in patients with DME who have good glycemic control and are undergoing treatment as needed.
